# Dual Retro-Aortic Left Renal Vein with Drainage into Left Common Iliac Vein: Rare Anomaly of Left Renal Vein

**Published:** 2016-05-01

**Authors:** H. C. Sutariya

**Affiliations:** Assistant Professor, Department of Radiology, G. R. Doshi and K. M. Mehta Institute Of Kidney Diseases & Research Centre (IKDRC)- Dr. H.L. Trivedi Institute Of Transplantation Sciences (ITS) Civil Hospital Campus, Asarwa, Ahmedabad- 380016, Gujarat, India

**Keywords:** Aorta, Transplantation, Nephrectomy, Renal veins, Iliac Vein

## Abstract

Knowledge of the renal vascular anatomy greatly contributes to the success of surgical, invasive and radiological procedures of the retroperitoneal region. In today’s era of transplant, this knowledge is of utmost importance in performing donor nephrectomy so that number of fatal intra-operative complications can be prevented. Herein, we report on a rare anomaly of left renal vein in which dual retro-aortic left renal veins were noted and one of them drained into the left common iliac vein.

## INTRODUCTION

Knowledge of the anatomy and its variations of renal veins is mandatory for retroperitoneal surgeries and venographic procedures. Variations in the origin, course and termination of renal veins mainly result from the anomalies of the embryological development. Normally, a single renal vein drains the left kidney. However, supernumerary left renal vein, retro-aortic left renal vein, and circumaortic left renal vein are other observed anatomical anomalies. Here, in our case, dual renal venous drainage was noted on left side coursing in retro-aortic location with one of them draining into the left common iliac vein.

## CASE REPORT

**Figure 1:**
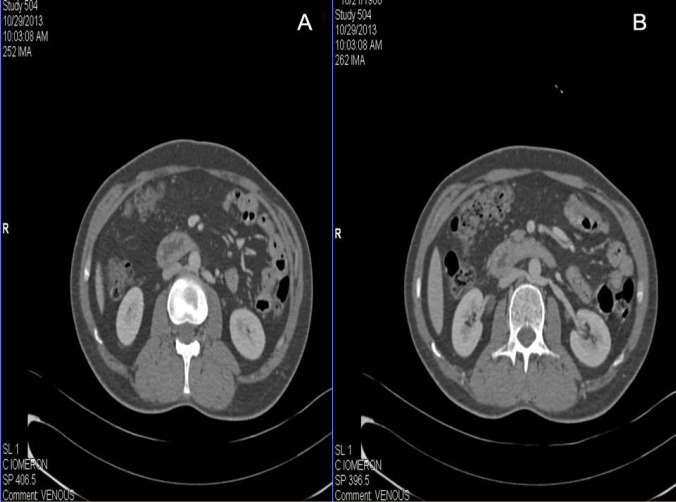
Axial images of the venous phase of CT renal angiography showing two left renal veins draining into the IVC with retro-aortic course

**Figure 2:**
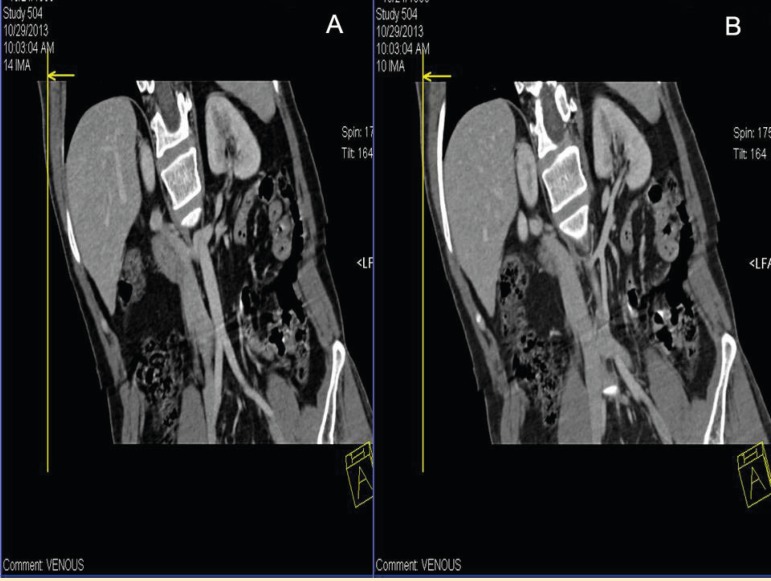
Coronal reformatted image of the venous phase of CT renal angiography showing two retro-aortic left renal vein with drainage into the IVC as well as the left common Iliac vein

**Figure 3:**
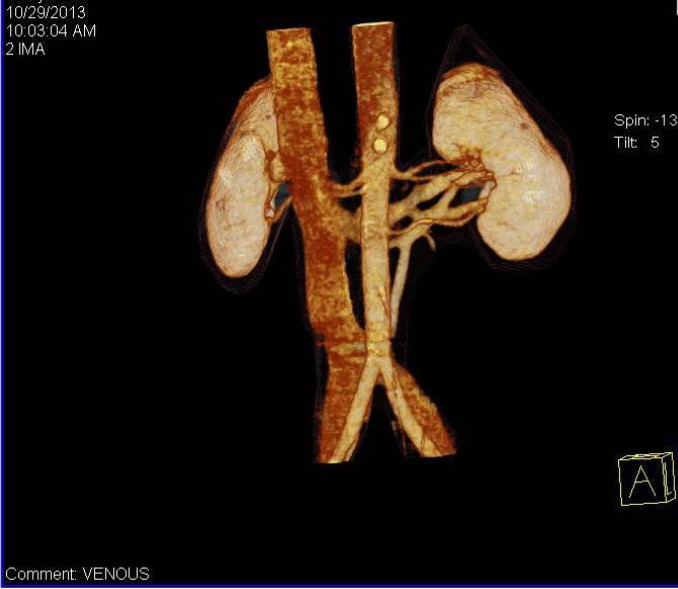
VRT image of retro-aortic left renal vein with drainage into the IVC as well as the left common iliac vein

A 46-year-old healthy male was evaluated as a potential renal donor for his son. In CT renal angiography, the dual renal veins were found on the left side with both of them coursing in retro-aortic location instead of normal pre-aortic location (Fig 1). One of the two left renal veins coursed oblique and drained into the lateral part of IVC; the other left renal vein coursed parallelly with the above-mentioned vein up to short length and then coursed downwards to drain into the left common iliac vein (Fig 2). The left adrenal vein drained into the left renal vein, which drained into IVC. Left gonadal vein and lumbar vein drained into the limb, which drained into left common iliac vein. Volume rendering technique reformation in CT scan also confirmed the drainage of both left retro-aortic renal veins (Fig 3). No other malformation, such as scoliosis of the spine or varicocele, was noted. The patient was finally taken for donor nephrectomy and operated without any intra- or post-operative complications. 

## DISCUSSION

Variations of renal veins are usually clinically silent and remain unnoticed until discovered during venography, operation, or autopsy. For transplant surgeons, morphology acquires special significance, since variations influence technical feasibility of operation. Variations restrict availability of vein for mobilization procedures [1]. The left renal vein passes horizontally between the abdominal aorta and the superior mesenteric artery to reach the IVC. The most common spinal level for drainage of renal veins is between the first and second lumbar vertebrae. The left renal vein is three times longer (7.5 cm) than the right and crosses anterior to aorta to open into the left lateral aspect of the IVC. A retro-aortic left renal vein is frequently encountered in radiological investigations. The incidence of retro-aortic vein is reported as high as 0.5% to 17% [1]. The course of the retro-aortic renal vein can vary considerably. It may drain into the IVC at the same level of its origin or more inferiorly. When the renal vein runs in retro-aortic location, as reported in this case, it is not uncommon for a lumbar vein to enter the renal vein that could complicate surgical procedures in the lumbar region. Lumbar vein drains into the left renal vein in 40% of cases [2, 3]. Various researchers have studied the incidence of retro-aortic left renal vein (Table 1) [4].

**Table 1:** The incidence of left retro-aortic renal vein as reported by other researchers [4]

Researchers	Incidence (%)
Pick and Anson (1940)	16.8
Chuang, *et al* (1974)	2–3
Reed, *et al* (1982)	1.8
Trigaux, *et al* (1998)	3.7
Bergman (2000)	1.5–8.7
Dhar and Ajmani (2004)	7.8
Tatar, *et al* (2008)	0.5–6.8
Anupama Gupta, *et al* (2011)	6.6

Retro-aortic left renal vein may be compressed between the aorta and the lumbar spine leading to left renal venous hypertension, which is known as posterior nutcracker syndrome, which is manifested by left flank and abdominal pain with or without hematuria.

Compression of retro-aortic left renal vein can cause left renal to gonadal vein reflux resulting in lower limb varices and varicocele, which may produce difficulties in spermatogenesis and may lead to infertility. It has been also associated with pelvic congestion syndrome in females manifested by lower abdominal pain; dysmenorrhea; dyspareunia; vulval, gluteal or thigh varices; and emotional disturbances. It may be obstructed by pressure from retroperitoneal growths leading to congestion of kidney and if long standing, it may give rise to a form of chronic interstitial nephritis.

The availability of the long left renal vein for mobilization procedures gets restricted and loses the advantages that normally occur from the greater length of the left renal vein. Moreover, the presence of the left retro-aortic renal vein bears clinical significance because of its susceptibility to injury during retroperitoneal surgery [4].

The anatomic knowledge of abdominal vasculature and its variations is of importance for a surgeon who approaches retroperitoneal region for various surgical procedures like renal transplantation, vascular reconstruction for congenital and acquired lesions, repair of abdominal aortic aneurysm, *etc*. Identification of retro-aortic left renal vein is very important in proper planning for nephrectomy, partial nephrectomy, and living donor nephrectomy. Failure to recognize this variant can lead to inadvertent injury and major venous bleeding. CT scan can detect this anomaly very precisely and can prevent any catastrophe.
